# Danger of an R-2 (Debulking) Cardiac Tumor Resection

**DOI:** 10.1016/j.jaccas.2025.103314

**Published:** 2025-04-09

**Authors:** Nitish Dhingra, Abdullah Ghunaim, Abdulaziz Alhothali, Dambuza Nyamande, Robert James Cusimano

**Affiliations:** Division of Cardiac Surgery, Toronto General Hospital, University of Toronto, Toronto, Ontario, Canada

**Keywords:** cardiac tumor, debulking surgery, R-2 resection, sarcoma

## Abstract

The natural history of cardiac tumors is dismal, with death ensuing rapidly. This is especially true with incomplete resection (called R-2 resection). We present a patient with metastatic sarcoma, also involving the heart, too unwell to undergo chemotherapy, who underwent a debulking operation, resulting in early rapid recurrence but response from chemotherapy. Debulking cardiac tumors may be selectively offered to enable chemotherapy initiation.

## Graphical Abstract

**Figure:**
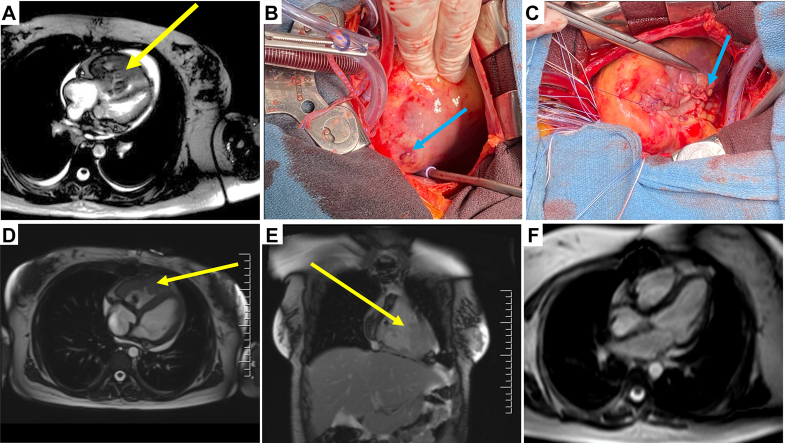

A 27-year-old woman with previously treated lower extremity osteosarcoma presented to hospital with a 1-month history of NYHA functional class IV dyspnea, anasarca, and pleuritic chest pain. A computed tomography scan revealed a right ventricular mass along with 2 right lower lobe nodules measuring 1.6 and 1.2 cm. Cardiac magnetic resonance demonstrated a severely dilated right ventricle (RV) with severe systolic dysfunction, and a large infiltrative heterogeneously enhancing mass occupying 90% of the RV (Figure 1A). Importantly, the patient also was noted to be in right heart failure, with a lactate of 8.6 mmol/L, platelets of 30 × 10^9^/L, bilirubin of 57 µmol/L, alanine aminotransferase and aspartate aminotransferase >1,000 U/L, and international normalized ratio of 3.2.

**Figure 1:**
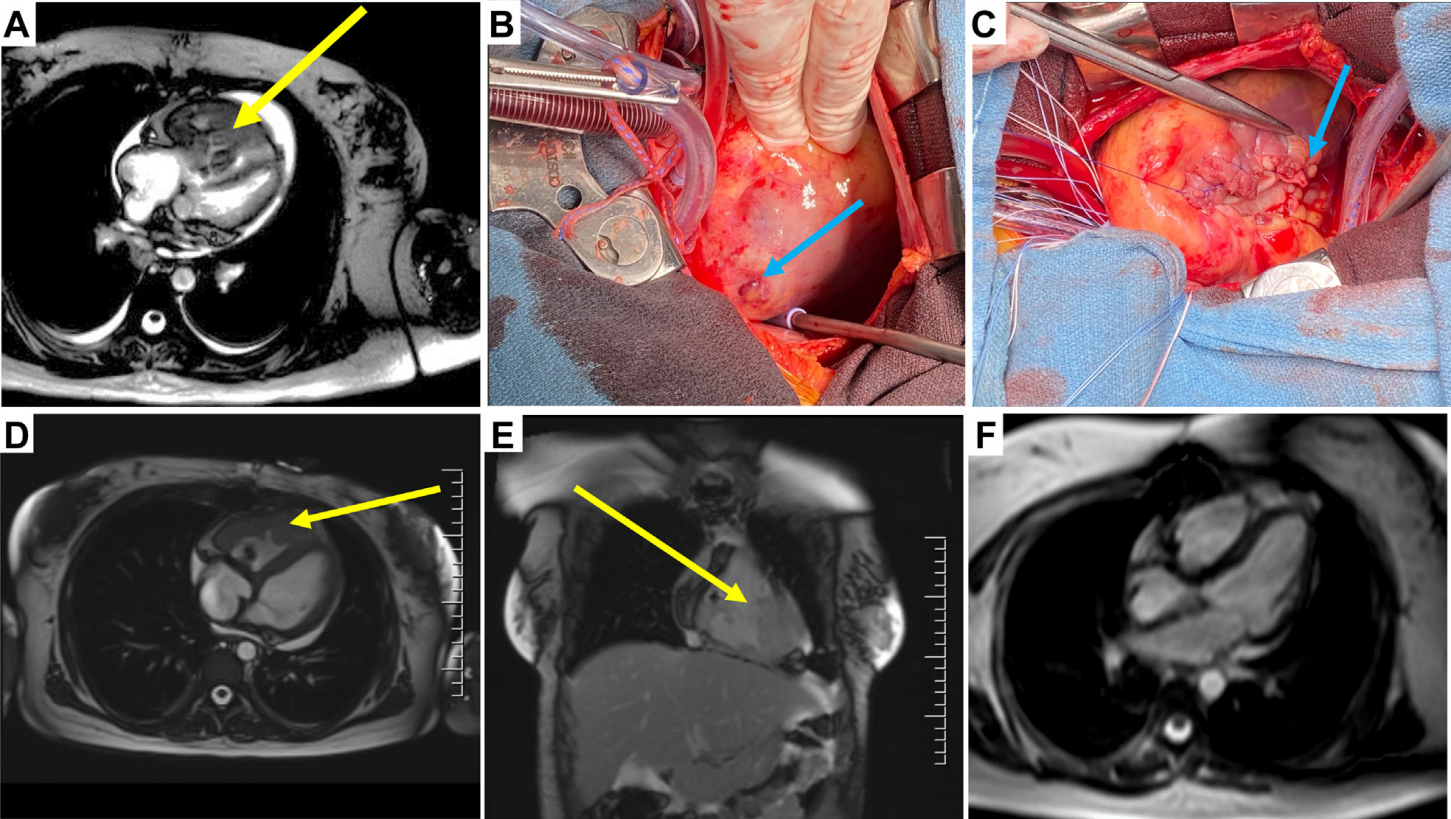
Radiological and clinical Tumor Images Serial cardiac magnetic resonance of metastatic sarcoma involving the right ventricle from preoperative (A), 2 weeks postoperative (D and E), and 3.5 years postresection and chemotherapy (F). Intraoperative images show penetration of the tumor past the surface of the inferior wall of the right ventricle (B) and after resection and reconstruction of the defect in the inferior wall of the right ventricle by bovine pericardium (C). Arrows depict findings on each panel.

The patient was originally seen by the medical oncology team for chemotherapy (doxorubicin/ifosfamide with dexrazoxane) for suspected recurrent metastatic sarcoma. However, given her heart failure, she was thought to be too unwell to tolerate chemotherapy. Cardiac surgery was consulted for resection to enable chemotherapy.

At operation, the tumor was noted to be eroding through the heart inferiorly (Figure 1B). The tumor was resected from the RV including a portion of the inferior wall; due to involvement, the tricuspid valve was replaced with a tissue valve (with subsequent warfarin therapy), and the inferior wall of the RV was reconstructed using bovine pericardium, impermeable and easy to work with (Figure 1C). There was very minimal residual tumor left at the right ventricular apex and along the septum (macroscopic, or R-2 residual tumor), but complete resection was thought to be impossible, and the goal was to allow enough resection to initiate systemic chemotherapy. The final diagnosis was sarcoma. A cardiac magnetic resonance performed 2 weeks later demonstrated very significant recurrence of the right ventricular tumor (Figures 1D and 1E). Importantly, however, the right ventricular outflow tract was unobstructed, and biventricular volumes were normal with mildly reduced global systolic dysfunction. After 2 cycles of chemotherapy, a treatment effect was documented with notable improvement in the bulky right ventricular free wall thickening. The patient’s most recent cardiac magnetic resonance approximately 3.5 years after surgery and chemotherapy demonstrated no evidence of recurrence or residual tumor in the RV, normal right ventricular free wall thickness, and normal biventricular size and function (Figure 1F).

Cardiac sarcomas remain a challenging clinical entity for diagnosis and management. With any cardiac tumor, the goal is to achieve complete microscopic resection (R-0) because this is thought to confer the best prognosis.^1^ Leaving any tumor behind is very deleterious to outcome. If contemplating an R-2 resection (debulking) for any malignancy, the patient must otherwise have a reasonable prognosis and adjuvant therapy must be started immediately postoperatively. Without this, recurrence and death may occur rapidly, often before sternal healing is complete. The rapidity of local recurrence demonstrated in this case shows how dangerous debulking can be without adjuvant therapy. The treatment provided was related to the presence of an obstructing tumor that prevented chemotherapy, not to the pathology. Operation alone is not meant to be a treatment, but rather an enabler for therapeutic treatment, especially with distant/systemic disease such as in this case. Unless care is multimodal and collaborative, rapid failure will ensue.

## Funding Support and Author Disclosures

The authors have reported that they have no relationships relevant to the contents of this paper to disclose.
